# Marked Improvement in Refractory TTP Directly after *H. pylori* Eradication Therapy

**DOI:** 10.1155/2016/1568586

**Published:** 2016-04-05

**Authors:** Irina Gringauz, Narin Nard Carmel-Neiderman, Tobin Mangel, Orith Portnoy, Gad Segal, Idan Goren

**Affiliations:** ^1^Internal Medicine Ward T, Sheba Medical Center, Tel Hashomer, Ramat Gan, Israel; ^2^Hasharon Hospital, Campus Rabin, Petah Tikva, Israel; ^3^Sackler School of Medicine, Tel Aviv University, Tel Aviv, Israel; ^4^St George's University of London, London, UK; ^5^Department of Diagnostic Imaging, Sheba Medical Center, Tel Hashomer, Ramat Gan, Israel

## Abstract

Thrombotic thrombocytopenic purpura (TTP) is a life-threatening disorder involving thrombotic microangiopathy and is characterized by increased platelet aggregation throughout the body. Acquired TTP can be triggered by a variety of conditions including infections. We hereby describe a case report of an 81-year-old female presenting to the internal medicine department with TTP and active chronic gastritis, positive for* Helicobacter pylori* (*H. pylori*) on biopsy. The TTP was highly resistant to medical therapy; however the patient underwent complete resolution of her TTP following* H. pylori* eradication. We conclude that acquired TTP may be triggered by* H. pylori* infection and that treating the underlying infection may play a role in improving TTP's outcome in some patients, especially when disease is refractory to medical therapy.

## 1. Introduction

Thrombotic thrombocytopenic purpura (TTP) is a life-threatening hematologic disorder involving thrombotic microangiopathy and characterized by platelet aggregation throughout the body. Acquired forms of TTP are known to be triggered by a variety of conditions including infections. We hereby describe a case report of an 81-year-old female presenting with refractory TTP with a subsequent complete resolution of her symptoms following eradication of* Helicobacter pylori* (*H. pylori*) infection.

## 2. Case Report

An 81-year-old female was admitted to an internal medicine (IM) department with a two-week history of petechial rash over her upper and lower extremities. The patient complained of recurrent headaches on a daily basis and profound fatigue and malaise during the past month. The patient denied having fever, any recent change in her regular medications, the use of herbal or over-the-counter medications, dietary changes, or recent travel. The patient also denied recent loss of weight, night sweats, hematuria, diarrhea, nausea, or vomiting. The patient had not noticed tarry stool and denied epistaxis. The patient's past medical history was significant for hypertension and osteoporosis. The patient's regular medications were a calcium channel blocker and hydrochlorothiazide and vitamin D with calcium supplementation. None of the patient's medications were changed in the past few years.

*Physical Examination and Laboratory Workup*. The patient's vital signs were normal, without fever. The patient was not in distress and did not have jaundice. Upon physical examination scattered petechiae were observed over the upper extremities. The rest of the patient's physical examination was within normal limits including heart, lungs, and abdomen. No hepato- or splenomegaly was noted and no melena or hematochezia was found in rectal exam. Initial neurological evaluation was within normal limits; however, during the patient's stay in the emergency room, new transient central facial nerve palsy appeared. Noncontrast enhanced head CT was negative for intracranial bleeding. Laboratory diagnostic tests revealed a white blood cell count of 6100 per microliter with 73% neutrophils, hemoglobin of 11.3 g/dL with a mean corpuscular volume (MVC) 94 femtoliters, and a reticulocyte count of 2.4%. Thrombocytes levels were 30,000 × 10^9^/liter. Schistocytes and spherocytes were identified in the peripheral blood smear. Coagulation functions and fibrinogen were all within normal limits. Blood chemistry revealed normal creatinine and liver function tests. Lactate dehydrogenase (LDH) levels were elevated at 405 IU/L. Serum haptoglobin was decreased (15.30 mg/dL). Workup for autoantibodies and the Coombs test were negative. ADAMTS13 activity test was less than 10% with positive ADAMTS13 antibodies. Bone marrow biopsy demonstrated slightly hypercellular marrow for the patient's age, with no other significant changes.

**Figure 1:**
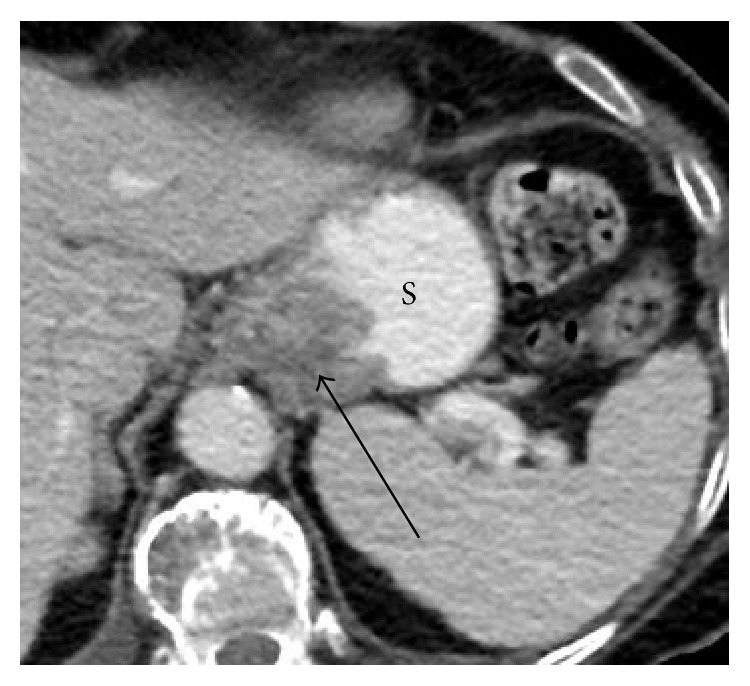
A CT slice of the left upper abdomen showing a large filling defect (arrow) with thickened folds at the cardia of the stomach (S).

*Clinical Course*. A diagnosis of TTP was made and the patient was started on plasmapheresis via a femoral catheter. Steroid treatment was initiated. In order to rule out underling malignancy a computed tomography (CT) scan of the chest and abdomen was performed and showed an intraluminal thickening of 3.5 cm size in the stomach (Figure 1). Further PET CT supported the findings demonstrating an unusual thickening of the gastric cardia with hypermetabolic activity in the distal third of the esophagus, suggesting a process with an increased metabolic activity.

**Figure 2:**
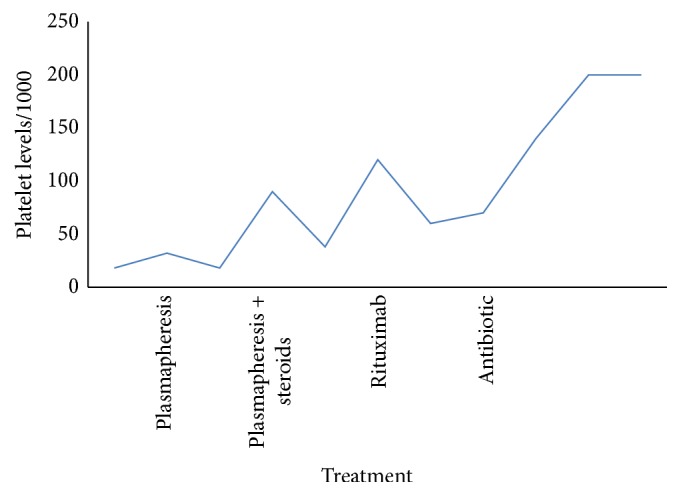
The graph illustrates the patient's platelet levels (per 1000) with different types of treatments. From the data, it is apparent that after the patient was given antibiotics the platelet levels were both stabilized and remained within normal limits.

At this stage rituximab treatment was initiated with only mild elevation in platelets count but no clinical significant improvement. During hospitalization the patient developed a deep vein thrombosis and treatment with low molecular weight heparin (LMWH) was initiated. Further investigation with gastroduodenoscopy demonstrated normal esophagus with a few erosions in the antrum of the stomach and was consistent with gastritis. Gastric biopsy found moderate active chronic gastritis and was positive for* H. pylori* and negative for dysplasia. Colonoscopy showed no significant findings. The patient was treated for 10 days with a triple-therapy regime consisting of omeprazole, amoxicillin, and clarithromycin. Soon after completion of antibiotics therapy TTP regressed and platelets count returned to normal (Figure 2). Similarly, both reticulocytes count and LDH levels were decreased after triple-therapy completion 2.5% versus 0.7% and 302 IU/L versus 182 IU/L for pretreatment versus posttreatment analysis accordingly. In addition, haptoglobin levels rose after eradication therapy from 31.2 mg/dL to 99.3 mg/dL. In the last follow-up one year later, the patient was well, without any complaints. Blood count and platelets remained within normal limits and the patient was free of symptoms.

## 3. Discussion

Firstly described by Moschowitz in 1924, thrombotic thrombocytopenic purpura (TTP) is a life-threatening hematologic disorder involving thrombotic microangiopathy and characterized by platelet aggregation throughout the body. The pathogenesis of acquired TTP is not fully understood but is believed to be mediated by autoantibodies that block the activity of ADAMTS13 enzyme leaving overactive large von Willebrand factor (vWF) multimers. The vWF multimers then initiate an enhanced degree of platelet aggregation. This in turn leads to the formation of microthrombosis in terminal arterioles and capillaries [1, 2].

Acquired forms of TTP are known to be triggered by various conditions including autoimmune diseases, malignancies, and infections [2]. In a review of 280 patients from France who were diagnosed with TTP, 41 percent had clinical features suggestive of an infection in the two weeks preceding the diagnosis or at presentation. Of those patients who were diagnosed with infection at the time of TTP diagnosis, 39.5% had infection involving the digestive tract and 21% were diagnosed during hospital stay with more than one infection [3]. The most widely described association between an infectious agent and TTP is that of Shiga toxin released from* E. coli*, predominantly types O157:H7 [4, 5].* E. coli* bacterial toxins are considered to be a main cause of TTP with no renal abnormalities in hemorrhagic colitis patients [5].* E. coli* toxins are also believed to decrease ADAMTS13 activity, thereby resulting in abnormally large von Willebrand factor multimers in plasma which in turn cause the disseminated platelet thrombi characteristic of TTP [6, 7].

Despite the high prevalence of infections prior to TTP presentation, only a few links between* H. pylori* active infection and TTP have been suggested. In a study published in 2005, a mechanism triggered by* H. pylori* infection was suggested as a pathogenic basis of an acquired form of TTP. This finding was based on a series of eight patients with TTP. The author proposed a possible interaction between the bacteria and vWF which initiates thrombocytes aggregation [8]. A similar relationship was found in patients after bone marrow transplantation. In a follow-up of 6 patients who developed TTP compared to 68 who did not, there was a significantly higher rate of* H. pylori* infection [9]. Taken together, our patient is the fifteenth patient in which the association between TTP and* H. pylori* has been found; however, more data is required to better establish this association.

## 4. Conclusion

We conclude that acquired TTP may be triggered by* H. pylori* infection, and therefore such examination is warranted in the evaluation of patients with TTP, especially when refractory to medical treatment. We suggest that by treating the underlying infection one can improve the outcome in patients with TTP who test positive for* H. pylori*, therefore promoting earlier TTP resolution.
